# Plasma Cholesterol Ester Fatty Acids: A New Biochemical Abnormality in Obstructive Jaundice

**DOI:** 10.1155/1995/82721

**Published:** 1995

**Authors:** M. W. Scriven, D. F. Horrobin, M. C. A. Puntis

**Affiliations:** 1 Currently Consultant Department of Surgery Wrexham Maelor Hospital Trust Clwyd Wrexham TX LL13 7 USA; 2 Efamol Research Institute Kentville Nova Scotia B4N 4H8 Canada; 3 Hepato-Pancreatico-Biliary Surgery Unit University of Wales College of Medicine Heath Park Wales Cardiff CF4 4XN UK

## Abstract

Changes in fatty acid patterns may explain many of the observed abnormalities found in obstructive jaundice. This study looked at fatty acids in plasma cholesterol esters, in a group of patients with obstructive jaundice and a matched group of controls. Significant abnormalities were demonstrated, most importantly a fall in essential fatty acids, in the jaundiced group. Overall the saturation of this fraction, as assessed by double bond index, rose. The essential fatty acids are important as factors in membrane function and as precursors of eicosanoids. The abnormalities found in this study provide further evidence of the significance of EFA in patients with obstructive jaundice.


*HPB Surgery*, 1995, Vol. 8, pp. 241–244
Reprints available directly from the publisher
Photocopying permitted by license only

© 1995 OPA (Overseas Publishers Association)
Amsterdam B.V. Published in The Netherlands
by Harwood Academic Publishers GmbH
Printed in Malaysia

### Plasma Cholesterol Ester Fatty Acids: A New Biochemical Abnormality in Obstructive Jaundice*

M. W. SCRIVEN**, D. F. HORROBIN[†] and M. C. A. PUNTIS

** Currently Consultant, Department of Surgery, Wrexham Maelor Hospital Trust, Wrexham, Clwyd, LL13 7TX
[†] Efamol Research Institute, Kentville, Nova Scotia, Canada B4N 4H8
Hepato-Pancreatico-Biliary Surgery Unit, University of Wales College of Medicine, Heath Park, Cardiff CF4 4XN, Wales

*(Received October 28, 1993)*

Changes in fatty acid patterns may explain many of the observed abnormalities found in obstructive jaundice. This study looked at fatty acids in plasma cholesterol esters, in a group of patients with obstructive jaundice and a matched group of controls. Significant abnormalities were demonstrated, most importantly a fall in essential fatty acids, in the jaundiced group. Overall the saturation of this fraction, as assessed by double bond index, rose. The essential fatty acids are important as factors in membrane function and as precursors of eicosanoids. The abnormalities found in this study provide further evidence of the significance of EFA in patients with obstructive jaundice.

KEY WORDS: EFA-essential fatty acids PUFA-polyunsaturated fatty acids MUFA-monounsaturated fatty acids SFA-saturated fatty acids DBI-double bond index 18C-EFA-18 carbon EFA 20C-EFA-20 carbon EFA

### INTRODUCTION

The underlying cause of the many abnormalities found in obstructive jaundice is still unclear. As outcome has been associated with many of the abnormalities, their aetiology is important. We have investigated many aspects of fatty acid status in obstructive jaundice. Our central hypothesis is that fatty acid abnormalities are responsible for the cellular dysfunction known to occur, in many systems, in obstructive jaundice. The reticuloendothelial system seems particularly important. The aim of the present study was to establish the pattern of fatty acids in an important plasma fraction, in patients with obstructive jaundice.

### MATERIALS AND METHODS

42 patients admitted for elective surgery, for benign conditions, made up the control group. None of these patients had any history of jaundice or other hepatobiliary disorders. Similarly, none had any condition or predisposition known to affect fatty acid patterns, such as atopy or diabetes mellitus. All had normal liver function tests.

The jaundiced group comprised 42 patients with extrahepatic biliary obstruction. 12 had bile duct gall stones and 30 had compression from tumours. 6 of the tumours were metastastatic-from stomach (2), breast (1),

* This work was supported by a grant from Scotia Pharmaceuticals, Woodbridge Meadows, Guildford, Surrey.

Elements of this work were presented at the 4th Congress of the World HPB Association, Hong Kong, June 1992. An abstract was subsequently published.

The pattern of plasma cholesterol ester fatty acids is abnormal in obstructive jaundice. Scriven MW, Stewart JCM, Horrobin, DF, Puntis, MCA. HPB Surgery 1992;5 (Suppl): 32.

oesophagus (1), rectum (1) and an unknown primary (2). The remaining 24 had pancreatic carcinomas (13), cholangiocarcinomas (8) ampullary carcinomas (2) and gall bladder carcinoma (1). None of the jaundiced patients had any other condition or predisposition known to cause fatty acid changes.

The two groups were well matched for age, sex and weight (Table 1). The mean plasma bilirubin for the jaundiced group was 247 micromol/l (range 39–540) and the mean plasma alkaline phosphatase was 823 IU/1 (range 233–3060). As stated previously, the liver function tests of the control patients were normal.

Venesection was undertaken in the morning, using a large bore needle without a tourniquet. These precautions were to try and minimise the potential effects of platelet aggregation and diurnal variation, on fatty acid patterns. Plasma was separated by centrifugation and stored at −70°C until analysis.

After thawing, lipid was extracted. The plasma cholesterol ester fraction was then separated and extracted, by thin layer chromatography. The methylated fatty acids in this fraction were then measured by fully automated packed-column gas liquid chromatography. The details of these methods have been described previously[1]. The results, as molar percentages, were then analysed using a microcomputer. To aid the interpretation of the results a number of variables were calculated. The use of these variables is necessary because no single fatty acid level gives an indication of fatty acid status. Their use has been reviewed (2). The variables include totals for groups of fatty acids, for instance total saturated fatty acids (SFA), and the double bond index (DBI). DBI is the mean number of double bonds per fatty acid. The n-6 and n-3 fatty acids constitute the essential fatty acids (EFA), which, in humans, are synonymous with the polyunsaturated fatty acids (PUFA). Statistical analysis was undertaken using two sample Student's t-tests.

**Table 1** Basic details of the patients in each group

| | *Sex* | *Age (median (range))* | *Weight (Kg) mean (95% CI)* |
|---|---|---|---|
| Controls patients | 23M:19F | 63 (37–87) | 70.8 (66.6–75.1) |
| Jaundiced patients | 23M:19F | 69 (22–88) | 67.8 (62.9–72.8) |

Using a two sample Student's t-test no statistical difference was found between the weights or ages of the two groups. 95% CI–95% confidence interval.

### RESULTS

The results are shown in Tables 2 and 3. The fatty acids in Table 2 are the major ones in the plasma cholesterol ester fraction-others are detectable but only in very small concentrations. In these tables the ratios of the mean results for the jaundice to control patients are given. The ratios for the calculated variables are shown graphically in Figure 1.

Similar analysis was undertaken to compare the 12 patients with jaundice due to gall stones, with the 30 with jaundice due to malignant disease. There was no significant difference (data not shown).

### DISCUSSION

Many abnormalities have been described in obstructive jaundice. Some, such as immune and reticulo-endothelial dysfunction, seem important in the major complications these patients suffer[3]. Others, in particular changes in plasma bile pigments and liver enzymes, are useful clinically. However, the fundamental cause of the poor outcome in these patients is still unclear.

**Table 2** Fatty acid results

| | *Controls Mean* | *95% Confidence Interval* | *Jaundiced Mean* | *95% Confidence Interval* | *p* | *Ratio of Jaundice: Control** |
|---|---|---|---|---|---|---|
| 16:0 | 10:82 | (10.04–11.61) | 11.05 | (10.59–11.51) | > 0.100 | 1.02 |
| 18:0 | 0.95 | (0.54–1.36) | 0.80 | (0.65–0.96) | > 0.100 | 0.84 |
| 18:1n–9 | 17.82 | (16.84–18.80) | 23.77 | (22.20–25.33) | < 0.001 | 1.33 |
| 18:2n–6 | 51.45 | (49.09–53.81) | 43.22 | (40.82–45.63) | < 0.001 | 0.84 |
| 20:3n–6 | 0.76 | (0.63–0.87) | 0.39 | (0.30–0.47) | < 0.001 | 0.51 |
| 20:4n–6 | 6.36 | (5.76–6.97) | 6.37 | (5.64–7.11) | > 0.100 | 1.00 |
| 20:5n–3 | 1.21 | (0.94–1.47) | 0.77 | (0.61–0.93) | 0.006 | 0.64 |
| 22:6n–3 | 0.94 | (0.43–1.45) | 0.51 | (0.38–0.63) | 0.100 | 0.54 |

*–Ratio of the mean for the jaundice patients to the mean for the controls
The results are shown as molar percentages. Statistical analysis by Student's t-test

**Table 3** Calculated variable results

| | *Mean* | *Group 1 : Controls 95% Confidence Interval* | *Mean* | *Group 2: Jaundiced 95% Confidence Interval* | *p* | *Ratio of Naundice: Control** |
|---|---|---|---|---|---|---|
| SFA | 12.31 | (11.16–13.46) | 12.33 | (11.78–12.88) | >0.100 | 1.00 |
| MUFA | 22.20 | (21.01–23.39) | 32.24 | (29.75–34.72) | <0.001 | 1.45 |
| EFA | 63.07 | (61.16–64.99) | 53.44 | (50.57–56.31) | <0.001 | 0.85 |
| DBI | 1.72 | (1.69–1.75) | 1.59 | (1.54–1.64) | <0.001 | 0.93 |
| Total (n–6) | 59.90 | (57.59–62.21) | 51.40 | (48.56–54.23) | <0.001 | 0.86 |
| Total (n–3) | 3.02 | (2.44–3.60) | 1.91 | (1.56–2.27) | 0.002 | 0.63 |
| 18C–EFA | 53.34 | (51.03–55.65) | 44.86 | (42.42–47.29) | <0.001 | 0.84 |
| 20C–EFA | 8.51 | (7.75–9.28) | 7.71 | (6.87–8.55) | >0.100 | 0.91 |

* –Ratio of the mean for the jaundice patients to the mean for the controls
The results are shown as molar percentages. Statistical analysis by Student's t-test
EFA-essential fatty acids, PUFA-polyunsaturated fatty acids, MUFA-monounsaturated fatty acids, SFA-saturated fatty acids, DBI-double bond index, 18C-EFA-18 carbon EFA, 20C-EFA-20 carbon EFA.

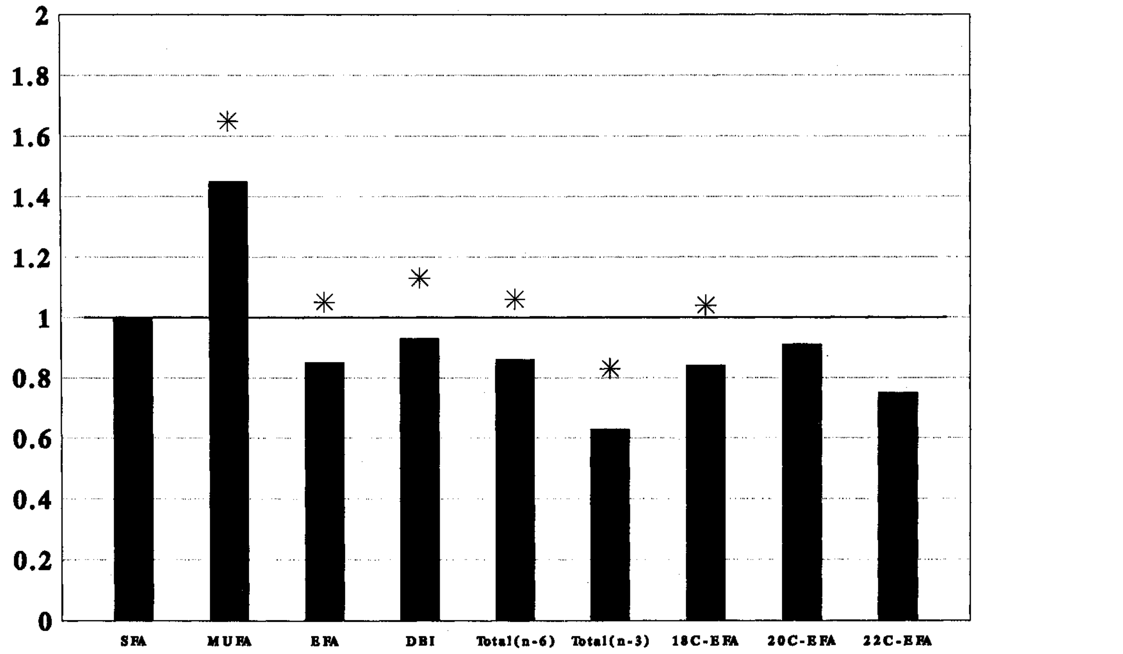

**Figure 1** The chart shows the ratios of the mean results of the jaundice to control patients, from Table 3. *- difference between the two groups statistically significant ($p \leq 0.002$). EFA-essential fatty acids, PUFA–polyunsaturated fatty acids, MUFA–monounsaturated fatty acids, SFA–saturated fatty acids, DBI–double bond index, 18C-EFA–18 carbon EFA, 20C-EFA–20 carbon EFA.

Fatty acids, in particular EFA, have important roles in the control of membrane function and as precursors of the eicosanoids[4,5]. Consequently, abnormalities in fatty acid patterns have profound effects on many systems. Reticuloendothelial cells are particularly sensitive to changes in fatty acid patterns[6–9]. The present study was part of a wider investigation of EFA in obstructive jaundice.

A number of highly significant abnormalities was shown. In summary there was a fall in EFA and a corresponding rise in monounsaturated fatty acids (MUFA), in the jaundiced group. The changes affected both n-6 and n-3 fatty acids. Of the three major eicosanoid precursors, eicosapentaenoic (20:5n-3) and dihomo-gamma-linolenic acids (20:3n-6) were lower than controls. Arachidonic acid (20:4n-6) was not different from controls. Overall, these changes were reflected as a fall in double bond index.

The direction of these changes is consistent with the hypothesis that fatty acid abnormalities underlie many of the changes in obstructive jaundice, especially those in reticuloendothelial function.

The aetiology of the fatty acid abnormalities was not investigated in this study. Possibilities include malabsorption of EFA and dysfunction of hepatic fatty acid desaturase enzymes. There was no difference between the patients with benign jaundice and those with malignant jaundice, indicating that biliary obstruction itself is more important than its cause.

A new biochemical abnormality has been demonstrated in obstructive jaundice. The changes are consistent with those demonstrated in other lipid fractions[10] and add to growing evidence of the importance of EFA in patients with obstructive jaundice.

### ACKNOWLEDGEMENTS

We are very grateful for the technical support of the Efamol Research Institute.

### REFERENCES

1. Manku, M. S., Horrobin, D. F. and Morse, N. (1983) Fatty acids in plasma and red cell membranes in normal humans. *Lipids*, **18**(12), 906–908.
2. Holman, R. T. (1986) Nutritional and functional requirements for essential fatty acids. *Prog. Clin. Bios. Res.*, **222**, 211–228.
3. Wardle, E. N. and Wright, N. A. (1970) Endotoxin and acute renal failure associated with obstructive jaundice. *Br. Med. J.*, **4**, 472–474.
4. Stubbs, C. D. and Smith, A. D. (1984) The modification of mammalian membrane polyunsaturated fatty acid composition in relation to membrane fluidity and function. *Biochem. Biophys. Acta.*, **779**, 89–137.
5. Smith W. L. (1989) The eicosanoids and their biological mechanism of action. *Biochem. J.*, **259**, 315–324.
6. Schroitt, A. and Gallily, R. (1979) Macrophase fatty acid composition and phagocytosis: Effect of unsaturation on cellular phagocytic activity. *Immunology*, **36**, 199–205.
7. Mahoney, E. M, Scott, W. A., Landsberger, F. R., Hamill, A. L. and Cohn, Z. A. (1980) Influence of fatty acyl substitution on the composition and function of macrophage membranes. *J. Biol. Chem.*, **255**, 4910–4917.
8. Schlager, S. I., Madden, L. D., Meltzer, M. S., Bara, S. and Mamula, M. J. (1983) Role of macrophase lipids in regulating tumoricidal activity. *Cell Immunol.*, **77**, 52–68.
9. Billiar, T. R., Maddaus, M. A., West, M. A., Curran, R. D., Wells, C. A. and Simmons, R. L. (1988) Intestinal gram negative bacterial overgrowth in vivo augments the in vitro response of kupffer cells to endotoxin. *Ann. Surg.*, **208**, 532–539.
10. Scriven, M. W., Stewart, J. C. M., Horrobin, D. F. and Puntis, M. C. A. (1991) Fatty acid metabolism is abnormal in obstructive jaundice. *Br. J. Surg.*, **78**, 1505.

